# Home Care Assistance: Has Covid-19 had an Impact on the Complex Management of HIV Patients?

**DOI:** 10.1007/s10461-022-03854-8

**Published:** 2022-09-26

**Authors:** Alex Dusina, Francesca Lombardi, Enrica Tamburrini, Fiorella Onorati, Massimo Petrucci, Simona Di Giambenedetto

**Affiliations:** 1 Fondazione Policlinico Universitario A. Gemelli IRCCS, UOC Malattie Infettive, 00168 Rome, Italy; 2 Dipartimento di Sicurezza e Bioetica Sezione Malattie Infettive, Università Cattolica del Sacro Cuore, 00168 Rome, Italy

**Keywords:** HIV, home care assistance, SARS-CoV-2, COVID-19, antiretroviral therapy

## Abstract

During the COVID-19 pandemic, people living with HIV (PLWH) could have had to face problems with treatment adherence because of the difficulty of accessing services connected with antiretroviral therapy (ART) dispensation, which could have undermined their health. In this article, we described, over the period 2015–2020, both the activities of our home care assistance unit, the “Unità di Trattamento Domiciliare (UTD)”, and the characteristics of the comorbid HIV patients followed-up. To determine whether the COVID-19 pandemic affected this service, we compared the number/type of services provided in 2020 with those provided in the preceding 5 years, i.e., 2015–2019. We also compared the proportion of monthly interventions carried out in 2018, 2019 and 2020. We found comparable values with some differences in the types of performances due to the heterogeneity of the population and their medical assistance needs. We also observed a stable viro-immunological status of the patients. All of these data suggest that the UTD was consistently active during the lockdown months and pandemic waves preventing therapy discontinuation, and was able to maintain optimal control of patients’ HIV infections.

## Introduction

Adherence to antiretroviral therapy (ART) is one of the key issues in patients living with HIV (PLWH). It is well known that optimal adherence to potent combination ART increases the likelihood of achieving viral suppression [1]; this, in turn, reduces HIV-associated morbidity and mortality, increases health-related quality of life and prevents HIV transmission [2, 3].

For some HIV-infected patients, being adherent to treatment is difficult. There are various reasons for lack of adherence: mental illness (such as depression), which is intrinsically associated with seropositive status, social stigma, comorbidities and the perception of treating an asymptomatic disease [4]. Some conditions strictly connected with HIV infection affect adherence to treatment. In particular, people who inject drugs have been indicated as a difficult-to-treat population, probably due to the association of this condition with psychiatric disorders and lack of social support [5].

The COVID-19 pandemic has had a significant effect on PLWH as it interferes with critical health services for HIV prevention, treatment and care [6]. This has been partly due to the difficulty of accessing services and ART during the pandemic [7] because of strict quarantine measures [8], shortages of ART because of lockdowns by certain drug manufacturers [9] and the reassignment of healthcare workers who normally provide care for PLWH to patients with COVID-19 [10, 11]. Some studies also suggest that COVID-19 has led to a higher rate of hospitalization, admission to intensive care units (ICU) and death of PLWH patients, particularly those with comorbidities (i.e., diabetes, renal failure and cancer) [12, 13]. To cope with the threat of insufficient adherence during the pandemic, healthcare systems had to adopt effective strategies to supply ART and assistance to both PLWH and patients with COVID-19, e.g., by adopting a multi-month ART dispensing policy and telemedicine platforms [7, 9].

In 1993, a home care assistance Unit, i.e., the “Unità di Trattamento Domiciliare (UTD)”, was established at our hospital, “Fondazione Policlinico Universitario A. Gemelli IRCCS”, with the aim of treating extremely complex or comorbid HIV-infected patients. This unit provides interventions, such as blood collection, intravenous infusion, drug delivery and administration directly at the patients’ homes in an attempt to overcome some of the barriers to treatment adherence. It also supplies medical advice and schedules radiological exams to permit easier access to hospital facilities.

The aim of this study is to describe the intervention of our UTD during the first year of the SARS-CoV2 pandemic and to determine whether there were any differences between the services provided in 2020 and those provided before the pandemic (i.e., 2015–2019).

## Methods

We retrospectively collected the data of patients followed by our UTD from 2015 to 2020. We selected this time frame for two reasons: first, data have been accurately collected by our UTD only since 2015 and the second-generation integrase inhibitor dolutegravir, one of the most common drugs used at our center, has only been available since that date. The inclusion criteria for this study were to be older than 18 years, being on ARV therapy and being followed by the UTD during the 2015–2020 time frame.

The UTD is managed by a physician who is supported by a resident and two nurses. It provides several services directly at the patient’s home. Here we have classified these services in five different categories: blood sample collection, dressing (skin wound and catheter), drug administration (intravenous infusion, intramuscular and subcutaneous injection), delivery of therapy (drugs and ART) and other services, i.e., adherence support, advanced wound care, electrocardiography, transfusions and aerosol therapy.

It also provides hospital assistance, i.e., medical appointment scheduling and accompanying patients to the hospital when necessary. This assistance covers an area in the north of Rome where our hospital is located. It covers 524Km^2^ and includes a population of 1,052,946 residents (medium-density housing of 2,009 inhabitants/Km^2^). It operates from 8.00 am to 2.00 pm from Monday to Saturday and provides services to five or six patients per day. The UTD service can take care of approximately 35 patients.

Usually the infectious disease specialists who care for HIV positive patients during their hospitalization or in the outpatient clinic refer to the UTD when a new, complex condition arises or when they discover a condition that can lessen patients’ compliance to therapy. These complex conditions can be clinical (i.e., neoplasia, bacterial infections that need intravenous/intramuscular therapy, the presence of skin wounds that require constant dressing), social (lack of other people who can help them manage their health problems, lack of economic means) or related to compliance (i.e., psychiatric disorders, difficulty reaching the outpatient clinic). The follow-up duration varies as it depends on the patients’ problems: some people are followed-up for only a few months if their condition improves or throughout their life if they are severely impaired. Visits are conducted at the patient’s home rather than the clinic. Specifically, the frequency of routine visits per patient is once a week. However, in the case of more complex patients, the UTD can provide up to two visits per week. During their routine visits, nurses can provide all the aforementioned services, i.e., one or more per visit, according to the patient’s needs.

Usually nurses can contact the physician by phone from the patient’s home at any time to resolve non-complex clinical problems. If a medical examination is necessary because of more severe issues, the physician goes directly to the patient’s home. Usually the physician carries out an in-person visit rather than a remote visit by phone about twice a month for each patient.

During these visits, the UTD manages clinical issues that are not only related to HIV infection (e.g., neoplasms and their complications) in interaction with other specialists in the hospital (i.e., oncologists, psychologists, psychiatrists and plastic surgeons).

In the context of the COVID-19 pandemic, specifically during the first lockdown (i.e., from March 9, 2020 to May 18, 2020), the activities were substantially the same with a few differences to optimize care and to reduce contact with patients and their families. Specifically, the UTD dispensed more than one ART drug pack at a time and the patients’ relatives were trained to administer therapy and manage wounds. This was true especially in the case of less complex patients. Moreover, the UTD tended to include more than one service per session (e.g., dressing wounds and blood collections) and also increased its phone support. Considering the overall percentage of services, we included about 20% of the services that were delivered by the UTD in the hospital for safety reasons (i.e., fewer contacts with possibly infected patients, greater availability of personal protective equipment, adherence to lockdown measures), especially for those autonomous patients who were able to reach the hospital easily.

We considered virological failure as two consecutive viral loads > 50 copies/mL after reaching viral suppression or the inability to reach viral suppression 24 weeks after beginning effective ARV; blip as a single viral load > 50 copies/mL; virological suppression as a confirmed HIV RNA level < 50 copies/mL [14].

We performed a descriptive analysis of the main characteristics of the population under the care of the UTD each year and the number/type of interventions made according to the year. To compare categorical variables reported as percentages we used the Chi square test; to compare the continuous variables, reported as median values, we employed the non-parametric Kruskal-Wallis or Mann-Whitney test.

We evaluated whether there were any differences in the percentages of services provided during 2020 with respect to the previous 5 years (2015–2019) and we compared the percentage of monthly services provided in 2018, 2019 and 2020. For this study, data regarding viro-immunological, therapeutic and clinical parameters were obtained from the hospital electronic database. For cost management purposes, the UTD nurses collected clinical records to keep track of the number and type of services supplied. Data were coded and entered using Microsoft Excel 2010 and were subsequently analyzed with SPSS v. 23 (SPSS IBM Inc, Chicago, Illinois, USA). P values less than 0.05 were considered statistically significant.

## Results

From 2015 to 2020 the UTD actively followed a stable number of HIV-infected patients, i.e., ranging from 26 to 31 per year for a total of 101 patients. The characteristics of the patients according to year are summarized in Table 1. Specifically, the characteristics of the patients followed by the UTD in 2020 were the following: 13 subjects (i.e., 42%) were male with a median age of 53 (interquartile range, IQR 47–62) years. Twelve patients were HCV positive (39%). The most frequent comorbidities were heart disease (26%, i.e., myocardial infarction or chronic heart failure) followed by cancer (23%, i.e., both active cancers and previous ones), hypertension (19%), and psychiatric diseases (19%, particularly depression, anxiety and drug or alcohol addiction).

**Table 1:** Characteristics of HIV-positive patients followed by the UTD over the six-year study period

	2015	2016	2017	2018	2019	2020	P value	Test Statistic (χ2/H test)
**N of patients**	29	29	26	30	31	31		
**Male gender, n (%)**	19 (65)	17 (59)	14 (54)	17 (57)	15 (48)	13 (42)	0.549^a^	4.01
**Age, years, median (IQR)**	55 (49–70)	56 (46–71)	54 (48–58)	53 (44–58)	52 (45–60)	53 (47–62)	0.878 ^b^	1.78
**Risk factor for HIV, n (%)**							0.942 ^a^	7.51
**- MSM**	8 (28)	5 (17)	8 (31)	7 (23)	5 (16)	5 (16)		
**- Heterosexual**	11 (38)	12 (47)	12 (46)	12 (40)	11 (35)	10 (32)		
**- PWID**	6 (21)	8 (28)	4 (15)	7 (23)	10 (33)	10 (32)		
**- Other**	4 (13)	4 (13)	2 (8)	4 (16)	5 (16)	6 (20)		
**Schooling**							0.927^a^	11.67
**-Primary school**	2 (6.9)	1 (3.4)	1 (3.8)	1 (3.3)	2 (6.50)	0 (0)		
**-Secondary school**	8 (27.6)	10 (34.5)	15 (57.7)	12 (40.0)	12 (38.7)	10 (32.3)		
**-High school**	10 (34.5)	8 (27.6)	6 (23.1)	9 (30.0)	7 (22.6)	9 (29.0)		
**-Bachelor’s degree**	3 (10.3)	2 (6.9)	1 (3.8)	1 (3.3)	2 (6.50)	2 (6.5)		
**-Unknown**	6 (20.7)	8 (27.6)	3 (11.5)	7 (23.3)	8 (25.8)	10 (32.3)		
**Income**							0.525^a^	23.91
**-Middle-income job**	1 (3.4)	1 (3.4)	3 (11.5)	5 (16.7)	1 (3.2)	0 (0)		
**-Low-income job**	4 (13,8)	2 (6.9)	3 (11.5)	4 (13.3)	4 (12.9)	3 (9.7)		
**-Disability pension**	5 (17.2)	7 (24.1)	8 (30.8)	8 (26.7)	11 (35.5)	10 (32.3)		
**-Pension**	12 (41.4)	12 (41.4)	9 (34.6)	8 (26.7)	9 (29.0)	6 (19.4)		
**-Unemployed**	2 (6.9)	1 (3.4)	1 (3.8)	2 (6.7)	2 (6.50)	5 (16.1)		
**-Unknown**	5 (17.02)	6 (20.7)	2 (7.7)	3 (10.0)	4 (12.9)	7 (22.6)		
**Time since HIV diagnosis, years, median (IQR)**	18 (10–23)	19 (16–28)	17 (2–24)	18 (3–26)	19 (3–27)	23 (3–29)	0.687 ^b^	3.09
**AIDS event, (or CDC C), n (%)**	15 (52)	16 (55)	12 (46)	15 (50)	15 (30)	15 (48)	0.988 ^a^	0.59
**Nadir CD4 cell count, cells/mm**^**3**^, **median (IQR)**	77 (17–210)	84 (21–199)	65 (10–230)	119 (5-263)	117 (27–217)	72 (31–217)	0.981 ^b^	0.74
**Zenith HIV-RNA, Log**_**10**_ **copies/mL, median (IQR)**	5.03 (4.06–5.69)	4.83 (3.87–5.39)	4.85 (3.68–5.84)	4.75 (3.85–5.30)	4.83 (3.72–5.66)	5.04 (4.38–5.95)	0.651 ^b^	3.32
**HCV positive, n (%)**	8 (28)	8 (28)	5 (19)	7 (21)	9 (29)	12 (39)	0.383 ^a^	5.28
**HbsAg positive, n (%)**	2 (6)	2 (6)	-	1 (3)	-	2 (6)	0.597 ^a^	3.68
**Comorbidities, n (%)**								
**- Hypertension**	8 (28)	9 (29)	7 (27)	8 (27)	8 (26)	6 (19)	0.963 ^a^	0.99
**- Psychiatric disease**	8 (28)	7 (24)	6 (23)	8 (30)	6 (19)	6 (19)	0.972 ^a^	0.87
**- Cardiac pathologies**	8 (28)	7 (24)	8 (31)	8 (30)	7 (23)	8 (26)	0.988 ^a^	0.59
**- Neoplasia**	6 (21)	12 (41)	9 (35)	10 (33)	8 (26)	7 (23)	0.516 ^a^	4.24
**- Chronic renal failure**	6 (21)	3 (10)	4 (15)	4 (13)	2 (6)	2 (6)	0.523 ^a^	4.19
**- Diabetes**	2 (7)	3 (10)	2 (8)	5 (17)	3 (10)	3 (10)	0.886 ^a^	1.72
**Time on ART, years, median (IQR)**	15 (7–20)	16 (5–21)	13 (1–19)	14 (3–24)	14 (2–22)	23 (3–23)	0.557 ^b^	3.95
**CD4 cell count during the year, cells/mm**^**3**^, **median (IQR)**	363 (203–535)	362 (141–458)	310 (200–620)	319 (242–643)	381 (200–553)	340 (176–614)	0.904^b^	1.58
**CD4/CD8 ratio during the year, median (IQR)**	0.43 (0.26–0.76)	0.42 (0.22–0.71)	0.34 (0.21–0.70)	0.47 (0.30–1.02)	0.41 (0.29–0.77)	0.41 (0.41–1.19)	0.409 ^b^	5.06
**HIV-RNA during the year, copies/mL median (IQR)**	5.7 (1.0-35.5)	4.0 (0.1–16.0)	6.5 (0.0-29.1)	0.0 (0.0-17.6)	0.0 (0.0-11.5)	0.0 (0.0-36.1)	**0.020** ^b^	**13.35**
**Naïve, n (%)** ^**c**^	1 (3)	2 (6)	2 (8)	1 (3)	3 (10)	3 (10)	0.841 ^a^	2.06
**Viral load > 50 copies/ml, n (%)**								
- **Virological failure**	4 (14)	2 (6)	6 (23)	2 (6)	2 (7)	2 (7)	0.236 ^a^	6.81
-**Blip**	5 (17)	5 (17)	1 (4)	-	2 (7)	1 (3)	**0.050** ^**a**^	**11.08**
**Death, n (%)**	3 (10)	5 (17)	5 (19)	5 (17)	2 (7)	-	0.152 ^a^	8.08
**Type of regimen, n (%)** ^**d**^							0.908 ^a^	4.73
- **Triple therapy**	22 (76)	20 (69)	17 (69)	20 (66)	21 (68)	21 (67)		
- **STR**	2 (7)	3 (10)	4 (15)	5 (17)	5 (16)	7 (23)		
- **Dual therapy**	5 (17)	6 (21)	5 (19)	5 (17)	5 (16)	3 (10)		
**Regimen containing, n (%)**							**0.018** ^a^	**21.45**
- **INSTI**	9 (31)	13 (44)	14 (54)	19 (63)	21 (68)	25 (80)		
- **PI**	15 (52)	12 (41)	8 (31)	8 (27)	5 (16)	3 (10)		
- **NNRTI**	5 (17)	4 (14)	4 (15)	3 (10)	5 (16)	3 (10)		
**Reasons for switch from previous therapy, n (%)**							**0.014** ^a^	**29.5**
- **Failure**	3 (10)	4 (14)	-	-	-	-		
- **Toxicity**	9 (31)	3 (10)	2 (8)	3 (10)	4 (13)	4 (13)		
- **Simplification**	6 (21)	8 (28)	6 (23)	10 (33)	10 (32)	14 (45)		
- **Other (such as drug interactions) /unknown**	11 (38)	14 (48)	18 (65)	17 (57)	17 (55)	13 (42)		

**Abbreviations:** MSM, Men who have sex with men; PWID, People who Inject Drugs; ART, Antiretroviral therapy; STR, Single Tablet Regimen; INSTI, Integrase strand transfer inhibitor; NNRTI, non-nucleoside reverse transcriptase inhibitor, PI, protease inhibitor. CDC stage C refers to the presence of an AIDS defining condition at diagnosis as listed In “*1993 Revised Classification System for HIV Infection and Expanded Surveillance Case Definition for AIDS Among Adolescents and Adults*”.

Bold/italics refers to significance at the p.

^a^ P value was calculated by Chi square test statistic (**χ2**).

^b^ P value was calculated by Kruskal-Wallis test statistic (H).

^c^ ART- naïve at the time of UTD program enrollment.

^d^ Type of regimen refers to the number of pills.

We observed that during the study period the patients’ demographic characteristics tended to remain stable with small, mostly non significant changes: male gender decreased over the years (from 65% to 2015 to 42% in 2020) (χ^2^ = 4.01, p = 0.549). Men who have sex with men (MSM) as a risk factor halved (i.e., from 28% to 2015 to 16% in 2020), whereas those who inject drugs (PWID) increased (from 21% to 2015 to 32% in 2020) (χ^2^ = 7.51, p = 0.942). Furthermore, the types of regimens did not change (χ^2^ = 4.73, p = 0.908) even though the anchor drug differed from year to year (χ^2^ = 21.45, p = 0.018). In fact, in 2015 most patients (62%) were treated with a PI-based regimen. However, over the years the use of PI has decreased, reaching 10% in 2020. The use of NNRTIs has also tended to decrease over the years, i.e., from 17% to 2015 to a minimum of 10% in 2020. By contrast, with the introduction and increased use of INSTIs we have observed a growing number of patients who are treated with INSTI-based regimens, i.e., from 31% to 2015 to 80% in 2020. Another important difference can be observed in the reasons for switching strategy (χ^2^ = 29.50, p = 0.014). In 2015 and 2016 virological failure was present with a frequency of 10% and 14%, respectively, but in recent years there has no longer been the need to change therapy following a failure. Toxicity, the most prevalent cause for switching to another regimen in 2015 (31%), dropped to 13% in 2020; inversely, a proactive simplification, initially observed in 21% of patients, reached 45%.

When we considered the immunological parameters, we found that both median values of the CD4 cell count and CD4/CD8 ratio were stable over time (χ^2^ = 1.58 and 5.06, p = 0.904 and 0.409, respectively). Although the median viral load has remained under 50 copies/ml, an overall marked improvement has been observed, particularly in the last few years (χ^2^ = 13.35, p = 0.020). In detail, when we compared the median CD4 count, CD4/CD8 and viral load between 2019 and 2020 we found no significant differences (U = 350.0, 313,0 and 299.0, p = 0.986, p = 0.499 and p = 0.288, respectively).

**Fig. 1:**
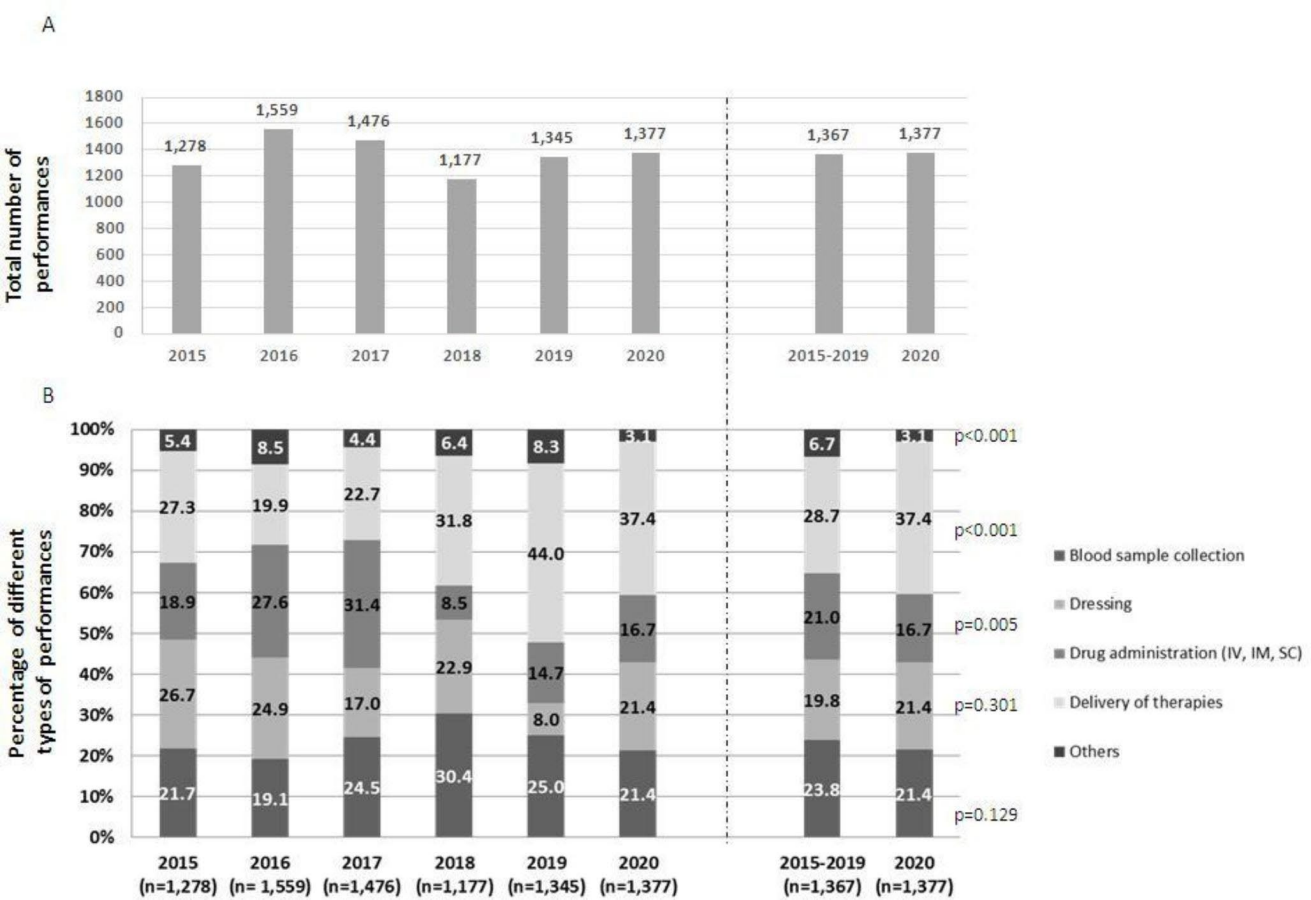
Services over the study period: (A) total number of services and (B) percentage of different types of services. Right panels present the direct comparison between 2015–2019 and 2020 (p values were calculated with the Chi square test)

The total number and type of main performances carried out by the UTD by year are summarized in Fig. 1 (A-B). The total number of performances carried out in 2020 was similar to the total number provided during the preceding year, i.e., 2019 (1,377 vs. 1,345). However, the number of interventions was different from year to year: in 2018, the UTD provided a minimum of 1,177 interventions and in 2016 a maximum of 1,559 interventions (Fig. 1 A). Given the fluctuation over the years, we also compared the total number of performances in 2020 with the mean of the total number of services carried out over the previous five-year period, i.e., 2015–2019, and found that they were superimposable (1,377 vs. 1,367) (Fig. 1 A, right panel).

When we considered in more detail the types of services provided by the nursing and the medical staff at patients’ homes in 2020, we found that they included collecting blood samples (n = 295, 21.4% of all services), dressing wounds (n = 295, 21.4%), drug administration (n = 230, 16.7%), delivery of therapies (n = 515, 37.4%) and others services (n = 44, 3.1%) (Fig. 1B). We observed fluctuations in the type of services provided year by year. Then we compared the single performances in 2020 with their mean performances from 2015 to 2019. The blood sample collection and the wound dressing carried out in 2020 remained similar to that carried out from 2015 to 201 (21.4% vs. 23.8%, χ^2^ = 2.30, p = 0.129 and 21.4% vs. 19.8%, χ^2^ = 1.07, p = 0.301, respectively); however, there was a significant difference in drug administration (16.7% vs. 21.0%, χ^2^ = 7.99, p = 0.005), delivery of therapies (37.4 vs. 28.7%, χ^2^ = 23.59, p < 0.001) and other services (3.1% vs. 6.7%, χ^2^ = 19.35, p < 0.001). Given the superimposable number of total performances between 2020 and the five-year time period, the proportion of the specific performances tended to balance one another (Fig. 1B right panel).

**Fig. 2:**
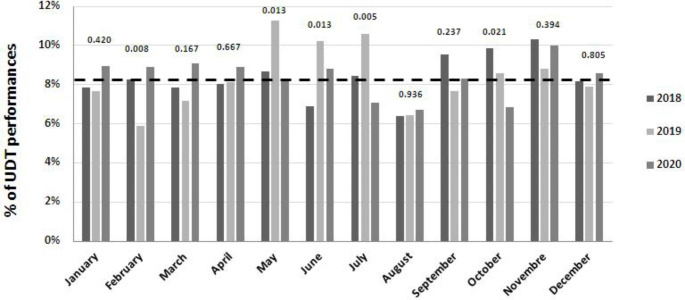
Comparisons among monthly percentages of services carried out in 2018, 2019 and 2020 (p values were calculated with the Chi square test)

Subsequently, we performed another analysis to compare the percentages of monthly performances in 2020 with those of the immediately preceding years, i.e., 2019 and 2018 (Fig. 2). In detail, we observed significant differences that were essentially related to the year 2019, which showed non constant percentages of performances month-by-month with the lowest peak in February (χ^2^ = 9.45, p = 0.008) and the highest peaks in May, June and July (χ^2^ = 8.63, 8.65 and 10.75, p = 0.013, 0.013 and 0.005, respectively). Instead, in 2018 and 2020 the percentages of services were more consistent over the months and remained at about 8.3% (the mean of the monthly performances); in August, which is a summer holiday month, the percentages of performances for all three years were similar, i.e., around 6.5% (χ^2^ = 0.13, p = 0.936).

## Discussion

In this study, we present a model to use in following-up complex HIV positive patients and in overcoming some of the barriers that prevent complete compliance, e.g., such as occurred during the COVID-19 pandemic and due to the lockdown restrictions. Investigation of the HIV-positive patients who frequent our healthcare facilities showed that interrupting or deferring outpatient visits and difficulty obtaining medical care due to the lockdown restrictions triggered feelings of abandonment and perceptions of worsening quality of care. This negatively affected the psychological wellbeing (i.e., it created distress, anxiety and depression) of this vulnerable population [15, 16]. Other studies have reported that during the COVID-19 pandemic the number of missed visits increased, ART dispensation decreased and the number of HIV patients who were hospitalized due to COVID-19 increased [17].

During 2020, there was a lockdown from March 9th to May 18th during which all social and commercial activities were closed, travel by any means was limited unless urgent/health related issues were present and people were compelled to stay at home and work remotely. Thus, many wards in our hospital admitted only COVID positive patients and outpatient clinics and Day Hospital services reduced their activities and treated only urgent clinical problems (e.g., the presence of an active neoplasia that required chemotherapy). Our outpatient clinic also drastically reduced the number of routine visits and admitted only non postponable evaluations (e.g., patients with non COVID-related fevers; monitoring patients who were on antibiotic therapy for bacterial infections). Regarding HIV-positive patients, the physicians dispensed multiple ARV drug packs in a single solution and postponed the collection of blood samples for monitoring viro-immunological parameters for stable patients. If clinical problems arose that required an evaluation, the patients usually contacted the physician by phone or email to make an appointment or to obtain advice about possible solutions to their problem (e.g., the need to change ARV due to intolerance or side effects).

Here, we show that the UTD seems is an efficient means for following-up these long-term treated and comorbid patients and obtaining an improvement in their virological parameters over time while keeping their CD4 levels stable and optimal. In particular, during the COVID-19 pandemic, it was able to efficiently carry out interventions during the year of this novel pandemic and even during the lockdown months.

Based on results, we hypothesize that the UTD provided a comfortable assistance setting for the control of HIV infection even in patients who were more prone to stopping treatments. The viro-immunological parameters of the patients followed-up by the UTD in 2020 remained at an optimal and stable level, similar to those observed in 2019. This might suggests that keeping constantly in touch with patients helps them establish a positive therapeutic relationship with healthcare workers (i.e., nurses and physicians), thus increasing compliance.

The differences in the UTD’s performances associated with the oscillation in the number of total interventions were probably due to the differences among the patients admitted to this service and their needs from year to year. In fact, this population includes a heterogeneous group of people (mostly treatment-experienced HIV patients) with different comorbidities that require different types of follow-up. In particular, the larger number of performances in 2016 might partly be explained by the higher number of neoplastic patients treated (i.e., 41%) who are by definition more complex than other PLWH.

A decline in the number of interventions in March, April and May 2020 was to be expected due to the outbreak of the pandemic and the subsequent lockdown. Note that one of the nurses came down with COVID-19 at the end of March 2020 and was not replaced by another one because the staff was already involved in treating COVID-19 patients. Our data show that during the COVID-19 pandemic our service basically remained active as in previous years, providing medical and nursing assistance to HIV positive patients and helping them to access the ART and other services. In any case, the UTD optimized care and adopted some strategies that were aimed at reducing contact with patients and their families, but still consistently followed-up the patients. Despite the efforts to decrease contacts with patients, the stability of the services can be partially explained by the fact that in 2020 services that required the constant physical presence of the nurses at the patients’ homes (i.e., to dress complex wounds or carry out the infusion of intravenous drugs) arose while drug dispensation decreased. Furthermore, in the overall percentages of services we need to consider that about 20% of the performances that were delivered by the UTD in the hospital were done so for safety reasons.

Some changes observed in the epidemiological characteristics from 2015 through 2020 might be explained by considering that the UTD is a dynamic reality and that some patients are discharged when their clinical conditions improve (i.e., neoplastic patients who stop chemotherapy, HCV-positive patients who achieve HCV eradication), some patients die or new complex patients are admitted to the service. Furthermore, some patients might have completely different needs over time. For example, the increasing presence of PWIDs could be due to the fact that they are more easily eligible to be followed-by the UTD because they are more prone to being affected by multiple pathologies (i.e. psychiatric diseases, HCV infection) and to worse compliance [18]. The increasing use of an INSTI-based regimen is most likely due to the availability of novel and more potent drugs (i.e., dolutegravir) that can be safely used also by complex HIV-infected patients. In fact, in PLWH affected by multiple comorbidities taking multiple drugs and who sometimes have compliance issues these last generation drugs and their new formulations (i.e. STR) are better tolerated, freer from significant drug interactions and toxicities and often allow reaching higher rates of virological suppression and possibly better immunological recovery [19].

This study has important limitations. First, due to the retrospective nature of the study the data are less standardized and more difficult to compare. There are also issues regarding the precise assessment of the patients’ needs each year and relating them to the differences in the services provided by the UTD. Also, our study did not take into account patients’ self perception of the services provided by the UTD or of their own compliance with ARV. Another limitation of our work is the lack of a comparator group. In any case, it was difficult to make a comparison between patients followed by the UTD and subjects attending our outpatient clinic because these settings are extremely different in terms of numbers and characteristics; in fact, the patients followed-up by the UTD are less compliant and more comorbid.

Despite these limitations, this study testifies to the fact that has been crucial to maintain the HIV care continuum during the COVID-19 pandemic with particular efforts to ensure timely access to care and to avoid the disruption of routine HIV services.

## Conclusion

In conclusion, we can see how this kind of home care assistance unit has been effective in preventing the discontinuation of therapy in complex, comorbid and long-treated HIV-infected patients even during this particularly difficult year in which pathologies other than SARS-CoV2 infection were neglected.

If these patients are not constantly followed-up and supported, they have a greater risk of experiencing distress and mental health issues and are more prone to showing compliance issues and the onset of other diseases related to poor control of HIV infection. In this scenario, creating a dynamic and constant presence like the UTD in the life of these patients could be a strategy for bridging the gap between providers and HIV-infected patients.

## Data Availability

All of the data used in this study will be made available upon request.
